# Prevalence, the antibiogram and the frequency of virulence genes of the most predominant bacterial pathogens incriminated in calf pneumonia

**DOI:** 10.1186/s13568-020-01037-z

**Published:** 2020-05-29

**Authors:** Abdelazeem M. Algammal, Mahmoud E. El-Sayed, Fatma M. Youssef, Shefaa A. Saad, Mahmoud M. Elhaig, Gaber E. Batiha, Wael N. Hozzein, Madeha O. I. Ghobashy

**Affiliations:** 1 Department of Bacteriology, Immunology, and Mycology, Faculty of Veterinary Medicine, Suez Canal University, Ismailia, 41522 Egypt; 2 Department of Clinical Pathology, Animal Health Research Institute, Ismailia, 41522 Egypt; 3 Department of Animal Medicine (Infectious Diseases), Faculty of Veterinary Medicine, Suez Canal University, Ismailia, 41522 Egypt; 4 Department of Pharmacology and Therapeutics, Faculty of Veterinary Medicine, Damanhour University, Damanhour, 2251 Egypt; 5 Bioproducts Research Chair, Zoology Department, College of Science, King Saud University, Riyadh, 11451 Saudi Arabia; 6 Botany and Microbiology Department, Faculty of Science, Beni-Suef University, Beni Suef, 62511 Egypt; 7 Microbiology Department, Faculty of Science, Ain Shams University, Cairo, Egypt

**Keywords:** BRD, Antibiotic resistance, *E. coli*, *S. aureus*, *P. multocida*, Virulence genes

## Abstract

The purpose of this study was to investigate the prevalence, antibiotic resistance and certain virulence genes of the most predominant bacterial pathogens causing BRD. A total of 225 calves; 55 apparently healthy and 170 diseased; were sampled. Bacteriological examination, antimicrobial susceptibility testing and PCR based detection of some virulence genes were performed. In addition, the serotyping of *E. coli* was performed using the slide agglutination test. The most predominant bacterial pathogens retrieved from apparently healthy calves were *E. coli* (16.4%) and *S. aureus* (10.9%), and in pneumonic calves were *E. coli* (23.5%), *P. vulgaris* (12.4%) and *S. aureus* (11.8%). The most prevalent virulence gene in *E. coli* was the *fim*H gene (100%), followed by *eae*A gene (24.5%) and *hly* gene (20.4%). All the examined *S. aureus* strains harbored *spa* and *coa* genes; likewise, all *P. multocida* strains harbored *tox*A gene. The majority of the isolated strains displayed remarkable sensitivity to norfloxacin and enrofloxacin; furthermore, the retrieved *E. coli* strains exhibited multidrug-resistance to gentamicin, erythromycin, streptomycin and trimethoprim-sulphamethoxazole, in addition, the isolated *S. aureus* and *P. aeruginosa* strains showed multidrug-resistance to amoxicillin, ampicillin and tetracycline. *E. coli* serogroups including O18, O143, O1, and O6 were retrieved from pneumonic calves as the first report in Egypt. In conclusion, the synergism between the conventional and genotypic analysis is an effective gadget for the characterization of bacterial pathogens causing BRD. Continuous surveillance of antimicrobial susceptibility is essential to select the drug of choice due to the development of multidrug-resistant strains.

## Introduction

Bovine respiratory disease (BRD) is one of the major risks to the health of calves and younger cattle, causing significant economic losses (Zeineldin et al. 2017). Viral infections, stresses, nutritional and environmental conditions are leading reasons for the commensal bacteria turned into to be opportunistic pathogens, leading to secondary respiratory infections and increase animal susceptibility to the respiratory disease (Clavijo et al. 2007; Griffin et al. 2010). Many bacteria are involved in BRD, including; *Staphylococcus aureus, Pasteurella multocida*, *Escherichia coli*, *Citrobacter spp.*, *Pseudomonas aeruginosa*, *Proteus vulgaris, Enterobacter aerogenes*, *Enterobacter cloacae*, *Bacillus spp.*, and *Mannheimia haemolytica* (de Oliveira et al. 2016; DebRoy et al. 2008; Timsit et al. 2017; Zeineldin et al. 2017).

Clinical signs of BRD vary from acute to chronic illness and include mostly high fever, depression, loss of appetite, cough, rapid respiratory rates, abnormal lung sounds and nasal discharge (Radostits et al. 2006). The utilization of antibiotics in the treatment of BRD has widely been reported. The emergence of multidrug-resistant pathogens associated with BRD has been recorded that threatens the livestock industry (Klima et al. 2014). The continuous surveillance of antimicrobial susceptibility should be applied as well as the proper use of antibiotics in order to reduce the spread of multidrug-resistant strains not only between livestock but also to humans (Holman et al. 2015; Klima et al. 2014).

The polymerase chain reaction (PCR) is a highly reliable and specific diagnostic tool that facilitates the detection of pathogens and their virulence genes as well as drug resistance genes (Eid et al. 2016; Enany et al. 2018). Therefore, the current work aimed to determine the prevalence of bacterial pathogens in apparently healthy and pneumonic calves and to monitor the antibiotic susceptibility of the isolated strains, and to perform PCR-based detection of certain virulence genes.

## Materials and methods

### Sampling

A total of 225 calves, in private cattle farms at the Ismailia governorate, Northern Egypt, were examined for respiratory manifestations from November 2018 to January 2019. The examined calves are ranging in age from one day to 6 months old. All calves were clinically examined calves were categorized into 3 groups, according to the clinical signs, the 1st group (*n *= 154) showed respiratory signs such as fever (> 39.5 °C), rapid respiration, coughing, and nasal discharge, the 2nd group (*n *= 16) showed only fever, and the 3rd group (apparently healthy; *n *= 55) showed no signs. Nasal swabs were gathered from 150 calves that exhibited respiratory signs and from 50 apparently healthy calves. Blood samples were collected in ethylenediaminetetraacetic acid (EDTA) from 16 calves that were showed only fever and from 5 apparently healthy calves. Four samples were collected from lung tissues of 4 emergency slaughtered calves. All specimens were collected and transported to the laboratory for further analysis.

### Bacteriological examination

The collected samples (nasal swabs and homogenized lung tissue) were inoculated into peptone water followed by incubation at 37 °C for 24 h. A loopful of incubated broth and blood samples were streaked onto Blood agar, Nutrient agar, MacConkey’s agar, Eosin methylene blue agar and Mannitol salt agar (Oxoid, UK), and then incubated at 37 °C for 24-48 h. The characterization of bacterial species was carried out based on the cultural characteristics, Gram’s stain, and biochemical reactions according to the previous report (Quinn et al. 2011).

### Serotyping of *E. coli*

Isolates of *E. coli* were serotyped based upon somatic and flagellar antigens using the slide agglutination test according to the previous report (Edwards and Ewing 1962). It was performed at Animal Health Research Institute, Dokki, Egypt, using a commercially available antisera kit (pathogenic *E. coli* immune sera) (Denka SeikenCo., Ltd., Tokyo, Japan). Bacteria showing agglutination within 30 s to antisera were estimated positive for the corresponding serotype.

### Pathogenicity test of the isolated *P. multocida* strains

The *P. multocida* strains were exposed to the pathogenicity test as the previously described protocol (Varte et al. 2014). Briefly, six mice were I/P inoculated with 100 μl of the whole culture; in addition, three mice were inoculated with 100 μl of sterile H_2_O (control group). All mice were observed for 2 days. Death within 24-48 h was monitored with postmortem examination. Re-isolation of *P. multocida* from the heart blood of dead mice was carried out by plating on blood agar. In addition, Giemsa stained smears from heart blood were made and microscopically examined. The handling of mice was carried out as described by the Animal Ethics Committee of Suez Canal University, Egypt.

### PCR detection of virulence genes

Genomic DNA of *E. coli*, *S. aureus* and *P. multocida* was extracted according to the manufacturer’s instructions of the QIAamp DNA Mini Kit (Catalogue No. 51304). The PCR reactions were done in a 25-μl volume containing 5 μl of 5× Master Mix (Jena Bioscience, Germany), 20 pmol of each primer and 3 μl of DNA template. Double-distilled H_2_O was added to complete the reaction volume to 25 μl. Positive controls kindly supplied by Animal Health Research Institute in Dokki, Cairo, Egypt and negative control (no DNA templates) were utilized in all PCRs. The primers sequences (Metabion International AG, Germany) and recycling conditions are listed in Table 1. The PCR products were separated by electrophoresis (1.5% agarose stained with ethidium bromide 0.5 μg/ml), and the amplicon was captured using a gel documentation system (Biospectrum UVP, UK).

**Table 1:** Primer sequences and PCR cycling conditions applied for the detection of virulence genes of *E. coli*, *S. aureus* and *P. multocida* strains isolated from examined calves

Bacterial Species	Target gene	Primer sequence (5′-3′)	Size (bp)	PCR cycling conditions (^°^C/s) for 35 cycles			References
				D	A	E	
*E. coli*	*fim*H	TGCAGAACGGATAAGCCGTGG GCAGTCACCTGCCCTCCGGTA	508	94/30	50/40	72/40	Ghanbarpour and Salehi (2010)
	*eae*A	ATGCTTAGTGCTGGTTTAGG GCCTTCATCATTTCGCTTTC	248	94/30	51/30	72/30	Bisi-Johnson et al. (2011)
	*hly*	AACAAGGATAAGCACTGTTCTGGCT ACCATATAAGCGGTCATTCCCGTCA	1177	94/30	60/50	72/60	Piva et al. (2003)
	*stx*1	ACACTGGATGATCTCAGTGG CTGAATCCCCCTCCATTATG	614	94/30	58/45	72/45	Dipineto et al. (2006)
	*stx*2	CCATGACAACGGACAGCAGTT CCTGTCAACTGAGCAGCACTTTG	779				
*S. aureus*	*spa*	TCA ACA AAG AAC AAC AAA ATG C GCT TTC GGT GCT TGA GAT TC	226	94/30	5/30	72/30	Wada et al. (2010)
	*coa*	ATAGAGATGCTGGTA CAGG GCT TCC GAT TGT TCG ATG C	430 570 630	94/30	55/40	72/45	Iyerand Kumosani (2011)
*P. multocida*	*tox*A	CTTAGATGAGCGACAAGG GAATGCCACACCTCTATAG	864	94/30	48/45	72/45	Tang et al. (2009)

*D* denaturation, *A* annealing, *E* extension

### Antimicrobial susceptibility testing

The antimicrobial susceptibility testing was carried out using the disc diffusion method on Mueller–Hinton agar (Oxoid, UK) according to the previous report (Wayne 2012). Erythromycin (15 μg), gentamicin (10 μg), tetracycline (30 μg), trimethoprim-sulphamethoxazole (25 μg), norfloxacin (10 μg), enrofloxacin (5 μg), streptomycin (10 μg), ampicillin (10 μg) and amoxicillin (20 μg) (Oxford, UK) were used. Results were estimated as described by the Clinical and Laboratory Standards Institute (CLSI 2016).

### Statistical analysis

The Chi square test was used for the analysis of the obtained results using statistical analysis (SAS^®^ software, version 9.4) to test the null hypothesis of various treatments.The significance level was (*p *< 0.05).

## Results

### Clinical examination

The clinical examination revealed pneumonia in calves approaching 68.4% (154/225), four calves died, and many cases showed mild calf scours. The farm record revealed respiratory infections, mortalities and scours a month prior to sample collection.

### Prevalence of bacterial pathogens in apparently healthy and diseased calves

Bacterial pathogens were isolated from 57% of the examined calves with or without BRD (Table 2). The statistical analysis revealed that there is a significant difference in the prevalence of various pathogens among the examined apparently healthy and pneumonic calves (*p* < .05). The most prevalent bacterial pathogen isolated from calves with BRD was *E. coli* (23.5%), followed by *P. vulgaris* (12.4%) and *S. aureus* (11.8%). Moreover, *E. coli* was the most prevalent bacterium isolated from apparently healthy calves (16.4%), followed by *S. aureus* (10.9%) and *P. vulgaris* (5.4%). In pneumonic calves, *P. multocida* (n = 3) was isolated from lung tissue samples and *M. haemolytica* (n = 1) was isolated from blood samples (Table 3). No bacterial pathogens were detected in the blood samples that were collected from the apparently healthy calves. The bacterial examination yielded 128 isolates (109 from pneumonic calves and 19 from apparently healthy calves). Moreover, 17 samples (7.6%) presenting a combination of two bacterial pathogens (mixed infection) (Table 4).

**Table 2:** The number and percentage of positive clinical samples using bacterial culture (BC)

Samples	Apparently healthy calves		Pneumonic calves		Total		*p*-value
	Samples, N	Positive samples, N (%)	Samples, N	Positive samples, N (%)	Samples, N	Positive samples, N (%)	
Nasal swabs	50	19 (36)	150	105 (70)	200	124 (62)	
Blood samples	5	0	16	1 (6.3)	21	1 (4.76)	< 0.00001*
Lung tissues (dead)	_	_	4	3 (75)	4	3 (75)	
Total	55	19 (34.5)	170	109 (64.1)	225	128 (57)	

*The result is significant at *p* < .05

**Table 3:** The prevalence of recovered bacterial pathogens from pneumonic and apparently healthy calves

Isolates	Source of sample	No. of isolates (%)	*p*-value
Pneumonic calves (*n* = 170)			
*E. coli*	NS	40 (23.5)	0.000118*
*P. vulgaris*	NS	21 (12.4)	
*S. aureus*	NS	20 (11.8)	
*P. aeruginosa*	NS	10 (5.9)	
*E. cloacae*	NS	7 (4.1)	
*P. multocida*	LT	3 (1.8)	
*E. aerogenes*	NS	7 (4.1)	
*M. haemolytica*	BS	1 (0.6)	
Total		109 (64.1)	
Apparently healthy calves (*n* = 55)			
*E. coli*	NS	9 (16.4)	
*S. aureus*	NS	6 (10.9)	
*P. vulgaris*	NS	3 (5.4)	
*E. aerogenes*	NS	1 (1.8)	
Total		19 (34.5)	

*NS* nasal swab, *LT* lung tissue, *BS* blood sample

*The result is significant at *p* < .05

**Table 4:** The frequency of mixed infection in the examined samples

Mixed isolates	Samples* (*n*, type)
Pneumonic calves	
*E. coli *+* E. cloacae*	5 (NS)
*E. coli *+* E. aerogenes*	5 (NS)
*S.aureus *+* Proteus vulgaris*	6 (NS)
Apparently healthy calves	
*E. coli *+* E. aerogenes*	1 (NS)
Total	17 (7.6%)

*Total number of samples (cases) = 225

### Serotyping of the isolated *E. coli* isolates

The isolated *E. coli* (n = 49) strains were examined serologically with O and H antisera. As described in Table 5, the serological analysis of revealed 8 serogroups in pneumonic calves; O18:H6 (n = 5), O119:H4 (n = 4), O86:H9 (n = 5), O158:H10 (n = 4), O143:H4 (n = 8), O1:H2 (n = 4), O63:H7 (n = 6) and O128:H2 (n = 4), and 3 serogroups in apparently healthy calves; O18:H6 (n = 4), O86:H9 (n = 3), O63:H7 (n = 2). The statistical analysis proved that there is no significant difference in the types of the O antigens of *E. coli* isolated from healthy calves and pneumonic calves (*p *> 0.05).

**Table 5:** The distribution of *E. coli* Serotypes in examined pneumonic and apparently healthy calves

Serotypes of *E. coli* (n = 49)	Pneumonic calves	Healthy calves	*p*-value
O18:H6	5	4	0.2^NS^
O119:H4	4	–	
O86:H9	5	3	
O158:H10	4	–	
O143:H4	8	–	
O1:H2	4	–	
O63:H7	6	2	
O128:H2	4	–	
Total	40	9	

NS: The result is non-significant at *p* < .05

### Pathogenicity test for *P. multocida* isolates

All the isolates of *P. multocida* killed all the tested mice (n = 6) 24 h after the inoculation. The postmortem examination showed generalized septicemia with congestion at subcutaneous tissues and internal organs. The stained smears from heart blood showed the characteristic bipolar pattern of *P. multocida.*

### PCR detection of virulence genes

As illustrated in Table 6, the *fim*H, *eae*A and *hly* genes were detected in 100%, 24.5% and 20.4% of the isolated *E. coli* strains (n = 49), respectively. However, *stx1* and *stx2* genes were not detected in the tested *E. coli* strains. Furthermore, all the examined *S. aureus* strains (n = 26) harbored both *spa* and *coa* genes (100% each). Additionally, the *tox*A gene was detected in all the tested *P. multocida* strains.

**Table 6:** The prevalence of virulence genes among *E. coli, S.aureus* and *P. multocida s*trains isolated from calves

Bacterial species (n)	Target gene	PCR positive, N (%)
*E. coli* (*n* = 49)	*fim*H	49 (100)
	*eae*A	12 (24.5)
	*hly*	10 (20.4)
	*stx*1	0
	*stx*2	0
*S. aureus* (*n* = 26)	*spa*	26 (100)
	*coa*	26 (100)
*P. multocida* (*n* = 3)	*tox*A	3 (100)

### Antimicrobial susceptibility testing

As illustrated in Tables 7 and 8, all the isolated *E. coli* strains were sensitive to enrofloxacin and norfloxacin (90% for each). Moreover, they exhibited multidrug-resistance against gentamicin (90%), erythromycin, streptomycin (80% for each) and trimethoprim-sulphamethoxazole (70%). Furthermore, the examined *S. aureus* strains showed remarkable sensitivity to enrofloxacin (90%), erythromycin, norfloxacin (80% for each) and gentamicin (70%), while showed multi-drug resistance pattern against amoxicillin, ampicillin (100% for each) and tetracycline (70%). In addition, all the examined *P. aeruginosa* strains were sensitive to enrofloxacin, norfloxacin and gentamicin, while showed multidrug-resistance against erythromycin, amoxicillin, ampicillin (100% for each) and tetracycline (80%). Regarding *P. multocia*, all the tested isolates were sensitive to gentamicin, enrofloxacin, and norfloxacin, while exhibited multidrug resistance against ampicillin, amoxicillin, erythromycin, and streptomycin (100% for each).

**Table 7:** In-Vitro antimicrobial susceptibility of the isolated bacterial pathogens from pneumonic calves

Antimicrobial agent	Disc conc.(μg)	*E. coli* (%) N = 40			*S. aureus* (%) N = 20			*P. aeruginosa* (%) N = 10			*P. multocida* (%) N = 3		
		R	I	S	R	I	S	R	I	S	R	I	S
Ampicillin	10	20	50	30	100	0	0	100	0	0	100	0	0
Amoxicillin	20	30	30	40	100	0	0	100	0	0	100	0	0
Erythromycin	15	80	20	0	20	0	80	100	0	0	100	0	0
Enrofloxacin	5	0	10	90	0	10	90	0	0	100	0	0	100
Gentamicin	10	90	10	0	20	10	70	0	0	100	0	0	100
Norfloxacin	10	0	10	90	10	10	80	0	0	100	0	0	100
Streptomycin	10	80	10	10	50	40	10	20	20	60	100	0	0
Tetracycline	30	40	20	40	70	10	20	80	20	0	0	33	67
Trimethoprim-Sulphamethoxazol	25	70	10	20	30	20	50	20	40	40	67	0	33

*Disc conc.* disc concentration, *S* sensitive, *I* intermediate, *R* resistant

**Table 8:** The multidrug-resistance patterns of the isolated bacterial pathogens from pneumonic calves

Antimicrobial agent	*E. coli* (N = 40), N (%)	*S. aureus* (N = 20), N (%)	*P. aeruginosa* (N = 10), N (%)	*P. multocida* (N = 3), N (%)
Ampicillin	8 (20%)	20 (100%)	10 (100%)	3 (100%)
Amoxicillin	12 (30%)	20 (100%)	10 (100%)	3 (100%)
Erythromycin	32 (80%)	4 (20%)	10 (100%)	3 (100%)
Gentamicin	36 (90%)	4 (20%)	–	–
Streptomycin	32 (80%)	10 (50%)	2 (20%)	3 (100%)
Tetracycline	16 (40%)	14 (70%)	8 (80%)	–
Trimethoprim-Sulphamethoxazol	28 (70%)	6 (30%)	2 (20%)	2 (67%)

## Discussion

This cross-sectional study analyzed the bacterial pathogens in healthy and pneumonic calves and provided a comprehensive analysis of the dominant bacterial pathogens causing respiratory infections in calves. The prevalence of causes of respiratory disease is highly changing within the cases and depends on various factors that affect the detected pathogens (Klima et al. 2014). Overall, the prevalence of bacterial pathogens in this study was 57%, comprising 62% of nasal swabs, 4.76% of blood samples and 75% of lung tissue (Table 2). These findings proved that the nasopharyngeal tract is a common habitat of opportunistic bacteria as well as the portal of entry of pathogenic infectious agents, which is endorsed by Holman et al. (Holman et al. 2015).

The most frequent bacterial pathogens isolated from the respiratory tract of apparently healthy calves were *E. coli*, followed by *S. aureus,* while in pneumonic calves were *E. coli, P. vulgaris* and *S. aureus*, these findings agree with previous studies characterizing the *Proteobacteria* in the nasopharyngeal tract of different animals (Holman et al. 2015; Ouchriah et al. 2015). Although de Oliveira et al. (de Oliveira et al. 2016) had isolated *E. coli* in a low proportion (4.22%) from healthy and diseased animals with respiratory signs, another report indicated the significance of *E. coli* in bronchopneumonia (DebRoy et al. 2008). The comparatively high proportion of *E. coli* in the study is worthwhile and of epidemiological importance. These findings suggest that these calves are likely to have originated from several farms and have a poor treatment and a negative immune deficiency and calls further characterization of more strains that will contribute to controlling the problem in local and/or international areas.

The bacterial variety of the bovine nasopharyngeal tract was much low when compared to other sites in the body (Holman et al. 2015). However, considerable different bacteria were observed in the present study from the nasal cavity of pneumonic calves greater than healthy calves as shown in Table 3, similar to previous reports (Angen et al. 2009; Clavijo et al. 2007; Ouchriah et al. 2015). Further, the finding of different bacterial pathogens in calves with pneumonia in this study supports the multifactorial causes of BRD in calves (Angen et al. 2009; Szeredi et al. 2010). A previous study revealed considerable variations in bacterial pathogens between healthy and diseased calves (Zeineldin et al. 2017).

In the current study, the *E. coli* serotyping revealed the serogroups; O86, O119, and O158, which were also demonstrated in diarrheic calves from Egypt by a previous report (Hakim et al. 2017). The serogroups; O18, O143, O1and O63 are specified in our survey and had a different variety than previous studies (Hakim et al. 2017; Vu-Khac and Cornick 2008), and to the best of our knowledge, this is the first report of these serogroups (O18, O143, O1, and O63) in *E. coli* strains identified from pneumonic calves in Egypt. Further, most *E. coli* strains isolated from healthy and pneumonic calves carry one to three virulence genes; *fim*H, *eae*A and *hly*. However, *stx*1 and *stx*2 genes were not detected, in agreement with a previous study (Osman et al. 2013). Extra-intestinal *E. coli* carried many virulent genes that have been detected in bronchopneumonia in horse (DebRoy et al. 2008). These findings showed that all *E. coli* strains isolated in the current study from nasal secretions of pneumonic calves are being pathogenic and may play a role in the epidemiology of BRD either alone or in combination with other pathogens. Also, *S. aureus* is one of the most common identified bacterial pathogens isolated from healthy and pneumonic calves (Table 3). All examined *S. aureus* strains harbored both *spa* and *coa* virulence genes. The proportion of *S. aureus* isolation in this study was higher than reported in calves (1.4%) from Brazil (Gaeta et al. 2018). This variation may be due to the difference in hygienic measures, farm management, and exposure to stress. Calves can inhale many environmental bacteria and can detect those bacteria in both the upper and lower respiratory systems, and therefore their opportunistic role should be looked (Gaeta et al. 2018; Griffin et al. 2010).

Although *P. multocida* is a significant agent of BRD (Griffin et al. 2010), in the current study, lower prevalence (1.8%) of *P. multocida* was recorded in diseased calves when compared to a prior report (5.5%) described by de Oliveira et al. (de Oliveira et al. 2016). A previous study indicated that *P. multocida* is frequently isolated from calves with pneumonia, which has previously been exposed to environmental and stress factors (Dabo et al. 2007). In addition, the virulence of *P. multocida* was confirmed in this study, where all tested mice have died 24 h later, which agrees with the results obtained by Okerman et al. (Okerman et al. 1979). Further, the *tox*A gene is a virulence gene associated with *P. multocida*, was identified by PCR in all isolates. A previous investigation reported that PCR targeting *toxA* gene is sensitive and essential for the detection of pathogenic *P. multocida* (Varte et al. 2014).

Moreover, *P. aeruginosa, E. cloacae, E. aerogenes,* and *M. haemolytica* were detected (Table 3) more frequently from diseased calves compared to the healthy calves. Previous studies recovered *Bacillus* species*, Klebsiella* species and *P. aeruginosa* in calves with bronchopneumonia (de Oliveira et al. 2016). Regarding pathogens, some species that are not of obvious importance associated with pneumonia, but their opportunistic role should not be ignored (Griffin et al. 2010).

Regarding the antibiogram of the most predominant isolates, as shown in Tables 7 and 8, most of the isolated *E. coli, S. aureus*, *P. multocida* and *P. aeruginosa* strains were highly sensitive to enrofloxacin and norfloxacin and showed variable degrees of resistance against the rest of the tested antimicrobial agents. The high sensitivity of enrofloxacin and norfloxacin is supported by previous reports (Kroemer et al. 2012; Kumar et al. 2009) and suggests that these antibiotics can still be utilized with a high chance of curative success for the treatment of respiratory diseases in bovines. Furthermore, the finding of high effectiveness of gentamicin against *P. multocida* is in contrast with a previous study on veal calves (Catry et al. 2005), who found that *P. multocida* was gentamicin-resistant. The multidrug-resistance was observed among the majority of the isolated *S. aureus*, *P. multocida* and *P. aeruginosa* strains against 3-4 antimicrobial agents, while most of *E. coli* strains were resistant to 4 antimicrobial agents, and could be attributed to the random use of antibiotics over time in animal production (Haftu et al. 2012) and of clinical significance, particularly in the calves industry system. Previous studies from Ethiopia, Brazil, Belgium, Egypt, and Canada have indicated that there is a remarkable increase in the prevalence of multidrug-resistant *E. coli, S. aureus* and *P. multocida* strains (Catry et al. 2016; Eid et al. 2019; Enany et al. 2019; Haftu et al. 2012; Timsit et al. 2017, Algammal et al. 2020).

In conclusion, the current findings described the bacterial causes of BRD in nasal swabs, blood samples, and lung tissues obtained from apparently healthy and diseased calves. *E. coli* and *S. aureus* were the most predominant bacterial pathogens incriminated in BRD. The lower prevalence of important pathogens like *P. multocida* may indicate the possible role of other types of opportunistic bacteria in BRD. To the best of our knowledge, *E. coli* serogroups; O18, O143, O1, and O63 were identified in cases of calf pneumonia as the first report in Egypt. Continuous surveillance of antimicrobial susceptibility is essential to select the drug of choice due to the development of multidrug-resistant strains. Enrofloxacin and norfloxacin were the most effective antimicrobials against calf respiratory pathogens. The application of both phenotypic and genotypic analyses is more valuable as a diagnostic tool for identifying the causes and could help in the proper treatment of BRD.

## Data Availability

Not applicable.
